# Implementing an evidence-based competency model for science team training and evaluation: TeamMAPPS

**DOI:** 10.1017/cts.2021.795

**Published:** 2021-06-08

**Authors:** Tiffany M. Bisbey, Kevin C. Wooten, Maritza Salazar Campo, Theresa K. Lant, Eduardo Salas

**Affiliations:** 1 Department of Psychological Sciences, Rice University, Houston, TX, USA; 2 Office of the President, University of Houston Clear Lake, Houston, TX, USA; 3 Institute for Translational Sciences, University of Texas Medical Branch, Galveston, TX, USA; 4 Department of Organization and Management, University of California Irvine, Irvine, CA, USA; 5 Department of Management and Management Science, Pace University, New York, NY, USA

**Keywords:** Team science, competencies, implementation, training, TeamMAPPS

## Abstract

**Introduction:**

In response to a call issued by the National Research Council to investigate the knowledge, skills, and attitudes of effective science teams, we designed a team training program for conducting science in collaborative contexts.

**Methods:**

We reviewed the literature to develop an evidence-based competency model for effective science teams along with exemplary behaviors that can be used for founding team training and evaluation. We discuss the progress of teamwork and team development research that serves as a foundation for this work, as well as previous research involving team-based competencies.

**Results:**

Three overarching competencies emerged from the literature as key for science team effectiveness: psychological safety, awareness and exchange, and self-correction and adaptation. These competencies are fully described, including their evidence base.

**Conclusions:**

We developed a competency model and implementation plan for a team training program specific to science teams – TeamMAPPS (Team Methods to Advance Processes and Performance in Science). This paper details steps in the implementation process, including plans for consortia dissemination, evaluation, and future development.

## Introduction

Recently, the National Research Council [1] issued a call to identify and translate the knowledge, skills, and attitudes (KSAs) that make science teams effective. Science teams are groups of scientists collaborating interdependently to conduct research. Team-based research allows scientists to put their expertise together toward solving complex issues more efficiently than individual efforts alone. [2, 3] To address important issues from all angles, science teams often employ a multidisciplinary approach to benefit from the insight of multiple perspectives. [4] Research suggests that multidisciplinary teams make a greater impact than individual scientists in producing academic and practical contributions, forecasting the steady increase in their prevalence to only continue. [5, 6]

Unfortunately, collaboration does not organically yield effective teamwork. As the scientific world is becoming more collaborative, researchers have begun to recognize a need to understand the teamwork competencies specific to the effectiveness of science teams, [7] as well as educational and training curricula for developing these competencies. [8] Working together effectively as a team requires specific KSAs that can be improved upon with team development interventions; [9] but it is currently unclear what KSAs might make science teams effective, as well as what barriers to effective teamwork are particular to this context.

Science teams need an actionable model to implement in team training to develop the competencies necessary for team effectiveness in producing and translating scientific findings. In this paper, we review existing competency models for teams in general and describe the development of an evidence-based competency model specific to science teams for use in training, development, assessment, and evaluation. In doing so, we contribute an evidence-based competency model complete with behavioral markers applicable to translational science teams at all levels of maturity. Further, we focus on the implementation process across multiple constituent groups.

We begin with a background of the field of team science, including existing models of teamwork and their applicability to science teams. Next, we discuss progress in the field of team development and evaluation to lay the groundwork for applying a new competency model for translational science teams. Finally, we detail the development of a competency model for science team effectiveness (Team Methods to Advance Processes and Performance in Science [TeamMAPPS]), and describe the implementation plan.

## Evolution of the Team Science Field

Research investigating team science began to emerge in the early 2000s. Problems were becoming more complex and ill-defined, requiring multiple areas of expertise and new collaborations to solve them. It was becoming clear that although the people collaborating can be experts in their own respective fields, that they may not necessarily make an expert team when collaborating together. Researchers were in need of evidence-based guidelines for collaborative science. [10]

There is an important distinction to make between teamwork and taskwork, as they each require unique skill sets. Taskwork skills are required for executing the specific tasks involved in completing work, and teamwork skills are required to collaborate with others and coordinate individual taskwork. [11] For instance, a pilot may pass all of their technical flight tests with a perfect score, but a plane will never leave the ground safely without appropriate skills for collaborating with the flight crew, such as closed-loop communication and conflict management. Teamwork skills are vital for effective collaboration in all fields, including conducting science. As is clear by the rarity of single-authored publications in academic journals, team science is now the norm across fields in academia, [12] medical science, [13] and translational science research, [14] and teams increasingly dominate solo authors in the production of new knowledge, producing more frequently cited research, and producing more high-impact research than individuals alone. [6]

As scientific, health, and societal problems are becoming increasingly complex in the 21st century, multiple disciplines and foundations of knowledge are needed to fully understand the issues and to develop solutions that address them from multiple angles. Accordingly, the National Science Foundation and the National Institutes of Health are increasingly requesting evidence of team science capacity as a criterion for awarding funding. Consider the recent COVID-19 pandemic – there is no single discipline able to solve such a crisis alone. Large-scale, complex issues require cross-disciplinary and cross-institutional level responses due to the sheer magnitude and threat they pose to the population. Our understanding of teams and the competencies required for effective teamwork is largely based on teams working in the same or similar discipline, for the same employer, in fields outside the realm of scientists creating new knowledge. Much of what we know about teamwork is general enough to apply to all situations, but there is a clear need to examine the challenges faced by scientific research teams specifically and to define the particular competencies that make them effective. It is both timely and necessary to advance a program for the training and development of translational science teams grounded in an evidence-based competency model for their effectiveness. [15]

## Status of the Team Training and Development Literature

Deficiencies and other barriers to team effectiveness are often addressed with countermeasures like team training and development. Reviews of team training [16] and meta-analyses [9] support a consistent relationship between teamwork training and team effectiveness. Research on science teams also suggests links between team effectiveness and the teamwork components targeted in team training. For instance, we know that science teams who develop shared mental models yield greater success, and that team member trust predicts the sharing of unique knowledge between science team members. [17] Investigations on team leadership in science teams find that transformational leadership plays a key role in fostering a climate for excellence and predicting innovation, [18] and it is positively related to changes in team development over time. [19] Moreover, team science leadership training can significantly influence leadership self-efficacy. [20]

Historically, work uncovering the competencies for evaluating team effectiveness unfolded alongside work on team training, stemming from multidisciplinary research in aviation, and the military. [21] From a more specific focus, researchers here [22] separated teamwork competencies into interpersonal KSAs (e.g., communication, conflict resolution) and self-management KSAs (e.g., goal setting, planning). Years later, researchers wondered if there was a “Big Five” of teamwork, of which all teams could be evaluated based on five core competencies, including: (1) effective leadership, (2) mutual performance monitoring, (3) backup behavior, (4) adaptability, and (5) team orientation. [23] A detailed list of teamwork competencies can be found in seminal efforts by Salas and colleagues, [24] with a recent update. [25] Recently, a Clinical and Translational Science Award (CTSA) endorsed project developed a working list of individual and team competencies for translational science, [15] which generated the competency domains of: (1) facilitating team affect, (2) team communication, (3) managing team research, (4) collaborative problem solving, and (5) team leadership.

The models afforded by existing work provide an excellent foundation for understanding the mechanisms that drive teamwork, but they require consideration of the work context in order for them to be useful in practice. The context and goals of a team dictate which competencies might be most important for any given team, as well as how key competencies might manifest. For instance, researchers in healthcare acknowledged the importance of effective teamwork for preventing medical error, [26] setting the forces in motion to eventually develop the TeamSTEPPS® training program specifically for healthcare teams. To develop TeamSTEPPS®, researchers [27] worked to understand the challenges that impact healthcare team effectiveness, then outlined the team competencies required to address those barriers to performance. The TeamSTEPPS® training program has had massive success improving the teamwork of healthcare providers across units and specialties, amassing an impressive evidence base as it continues to be implemented across the USA. [28] When it comes to science teams, no such fully delineated competency model exists to serve as a basis for team training and evaluation. Translational science teams have a unique set of goals and challenges that create a distinct context; and while we know much about what makes teams effective in general, we have yet to understand which teamwork competencies and behaviors are critical to address in science teams in the same manner that TeamSTEPPS® addresses healthcare teams.

## Challenges in Team Science Requiring Training and Development

Clearly, there is a long and impressive history of research demonstrating the effectiveness of teamwork across work contexts, as well as the impact of team training at improving team performance. Considerable literature exists concerning critical team-based KSAs, [24] as well as interdisciplinary team health research competencies. [29] This begs the question, *how are science teams any different?* In this section, we discuss the unique challenges facing translational science teams by highlighting three common characteristics that pose barriers to their effectiveness: (1) diverse backgrounds and skillsets, (2) distribution of teams or team members, and (3) membership and demographic changes over time.

### Diverse Backgrounds and Skillsets

One of the most salient characteristics of science teams is the diversity among members. As in virtually all teams and groups of people, diversity in gender, race, ethnicity, and other surface-level demographics can present hurdles for science teams to overcome. What is particularly notable in collaborative research is the characteristic presence of deep-level diversity in the functional backgrounds of team members. Because science teams are often deployed in response to complex questions posed by sponsors or in grant proposals, it is frequently the case for researchers to collaborate across disciplines, professions, and communities in effort to provide well-rounded and practically useful solutions considered from multiple perspectives; in fact, these collaborations are a stated requirement for many funding sources. [1] It is not always easy to collaborate with people who have different backgrounds or subscribe to different values, assumptions, norms, and expectations than one is accustomed to in their own domain. Nevertheless, it is important to have a variety of perspectives and expertise on a team, as demands fluctuate throughout the phases of any given project and different KSAs may be required for effectiveness. [30]

In addition, having different backgrounds means they may have different agendas outside of the science team, as well as hold different expectations for interactions within the operation of their respective silos. The cultural assumptions underlying behavioral norms and practices can vary widely across disciplines. Multiple perspectives means a lot of good ideas must be integrated, thus, more integration and translation may be required than in the typical brainstorming session. Moreover, scientists might be less accustomed to not being the only expert on their science team. Sharing leadership and responsibility might be difficult to maneuver as team members must set aside egos and learn to be flexible handing off leadership roles.

### Distribution of Team Members

As collectives grow in size, members can become more dispersed and siloed, exacerbating their differences and challenging their ability to act as a single unit. This is apparent at multiple levels, such as with departments across a university campus, large metropolitan areas with multiple universities, a country, global organizations, and the world itself. Although not all science teams are comparatively larger in nature, research suggests that their average size is increasing along with the impact of their collaborations. It is often the case that scientists collaborate across institutions to conduct research. [5] Unfortunately, large team size and dispersion of team members provide significant challenges to teams with regard to remaining coordinated, cooperating with one another, and maintaining shared mental models.

For instance, there are often struggles encountered in coordinating large groups of researchers in multi-institution projects, which essentially form teams within a larger team, or a multi-team system. [31] Such difficulties can require team members to ultimately set aside their differences to achieve their goals and produce important knowledge and practical implications, requiring significant effort to coordinate large teams across geographical and conceptual borders. [21]

### Membership and Demographic Changes

Science teams belong to no single discipline; thus, it may be rare for an individual’s core job duties to consist solely of their work on a particular research team. Rather, it may be more likely for team members to have responsibilities outside of their roles on the science team. Different priorities of team members and other common attributes of science teams (e.g., larger size, geographic distribution) can result in a considerable amount of time completing and progressing through stages of a project. During this time, the team might undergo various membership changes as project goals may change or different segments of the team become more or less important in different phases of the project. [30] Moreover, when distributed team members are highly interdependent, any changes in the team structure or other project conditions have the potential to impact the entire team and extend the timeline even further, as teams must regroup and revert back (to some degree) to a phase of socialization, trust building, and other activities that typically occur in the earlier stages of team development. Long timelines can provide significant challenges as a team-based research project develops, including greater chances for conflict, misunderstandings regarding members’ roles and responsibilities throughout project phases, difficulty remaining coordinated, and resistance to making necessary changes due to how long it takes to get things done.

## Results: Development of a Model for Science Team Competency – TeamMAPPS

Before developing a standardized team training program for science teams, it is essential to identify the core principles of teamwork that are essential to the performance context and the standards by which teams can be evaluated. Toward this effort, we reviewed the extant literature to substantiate an evidence base for a team training and evaluation program and a foundation for future research to build upon.

### Using Evidence to Develop the Model

In response to a call by the National Research Council, [1] we developed an evidence-based competency model for team scientists by identifying and translating KSAs for successful team science that can be applied to science team evaluation, training, and other educational curricula. Specifically, we developed a model that: (1) addresses known issues in team science, (2) is evidence-based, (3) can be described behaviorally, (4) can be used for development and/or evaluation, (5) can be applicable to nascent or advanced teams, and (6) can be used as an intervention and/or training model.

**Fig. 1.**
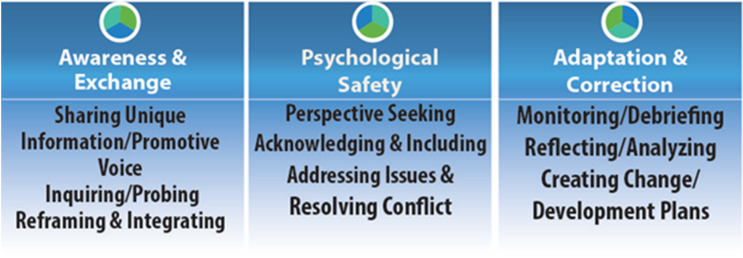
Specific components of TeamMAPPS. *Note*: TeamMAPPS = Team Methods to Advance Processes and Performance in Science.

Our model – TeamMAPPS – was developed on the basis of established team competencies and teamwork models applied to team science and to the scientific enterprise. Here, we used general team competencies, virtual team competencies, along with the core components of teamwork, and models of team behavior. A review of the team science literature and the Team Science Toolkit, [32] as well as the use of a comprehensive literature review [16] led to the logical development of three broad competency sets (Fig. 1).

Each competency is associated with an observable behavior, allowing for observational checklists that can inform evaluation plans and enable assessment when the behaviors are being practiced (Table 1). It is well established that training improves team-based competencies, including participant attitudes and the ability to integrate unique knowledge from others. To this extent, the TeamMAPPS would cover a number of the attitudes, behaviors, and cognitions required for effective teamwork. [16]

**Table 1.** Behavioral markers for TeamMAPPS competencies

Training Module	Competencies	Exemplary Behavioral Markers	Relevant Citations
Facilitating awareness & exchange	Sharing unique information/promotive voice	• Team members share information and perspectives stemming from their expertise • Team members share vision of team purpose • Team members build on others' ideas, but from their own perspective	Liang, Fahr, & Fahr, 2012; Salazar, Lant, Salas, & Fiore, 2012
	Inquiring/probing	• Team members inquire to gain information about who knows what in the team • Team members actively probe to create understanding that reflects inputs from all members	Huber & Lewis, 2010; Marks, Mathieu & Zacarro, 2001
	Reframing and integrating	• Team members report having been influenced by input from others • Team members integrate to create a shared vision of team purpose	Van Der Vegt & Bunderson, 2005; Klein, 2005
Promoting psychological safety	Perspective seeking	• Team members ask one another questions regarding their background • Team members provide vivid description of events that occurred • During team conversations, team members ask one another follow-up questions	Edmondson, 1999; Grant & Barry, 2003
	Acknowledging and including	• Upon hearing a new idea or opinion, team members rephrase the suggestion and acknowledge receipt of the message • New ideas are not criticized • Team members ask one another to step in when their skill set aligns with what tasks need to be completed	Edmondson, 1999
	Addressing issues and resolving conflict	• Errors and mistakes are openly discussed • Interpersonal conflict is minimized during group discussions • Team members are not criticized for making a mistake	Edmondson, 1999
Self-correction & adaptation	Monitoring/debriefing	• Team members adjust tasking to accommodate other team members' needs • Team members discuss performance episodes upon project/task completion • Incidents are documented	LePine, 2003; Tannenbaum & Cerasoli, 2013
	Reflecting/analyzing	• Documented incidents are reviewed and discussed by all team members • The team receives 360° feedback reports on performance • Team strengths and weaknesses are discussed among team members	Tannenbaum & Cerasoli, 2013
	Creating change/development plans	• Goals are discussed with and explained to all team members • Changes in work are completed without hesitation • Team members relearn procedures as necessary	LePine, 2003

*Note*: TeamMAPPS = Team Methods to Advance Processes and Performance in Science.

### Overall Framework of the TeamMAPPS Model

Based on our literature review of existing evidence, we have extracted three “competency sets” for the purpose of training: (1) awareness and information exchange, (2) psychological safety, and (3) adaptation and correction. Each competency set has three exemplary behaviors associated with them that will guide the assessment of the learning program (Fig. 1). Briefly, awareness and exchange is a set of competencies that facilitate team members’ unique voice, disciplinary insight, reframing and integrating alternative cognitive views; psychological safety is a set of competencies that provide a safe environment for different perspectives, including participant acknowledgement, issue identification, conflict resolution; and adaptation will result in team members being able to respond to external challenges, monitor the team and improve its performance through self-correction, and focus on development long-term.

### Awareness and Information Exchange

Because science teams are often assembled of expert team members in their own respective areas, it is essential for teammates to have an awareness of where expertise lies in the team. This awareness serves as the basis of the team’s transactive memory system (TMS). A TMS is the cognitive structure within a team through which they store, retrieve, and share information. It details who knows what and where to go for specific knowledge, and thus, serves an especially crucial role in teams with diverse skillsets. Research shows that high-performing teams are more likely to have high-quality TMSs that allow them to share knowledge quickly and effectively. [33]

Science teams can vary in the degree to which their knowledge is shared or unique, and their effectiveness hinges on their ability to integrate their unique knowledge into an effective strategy. [34] Part of this process may involve probing each other for relevant input so that expertise is included from all key areas. Effective science teams begin to develop an understanding of everyone’s areas of expertise by inquiring until enough information is shared to develop a shared idea of the team’s abilities. Once that knowledge is shared, it may need to be reframed in the context of the group’s goals so that it can be integrated into an effective performance strategy. [35] Expert science teams can draw upon the knowledge of each team member to create a shared vision and purpose for the team that guides goal-setting and strategy development.

Although much of the process of developing an awareness involves exchanging information, it does not mean that communication must be constant; rather, it is important for information which is unique to be shared and incorporated into the project plan. Moreover, it highlights the importance of using promotive voice to consistently improve strategies. The concept of voice in the workplace refers to expressing ideas, suggestions, opinions, or concerns about work. Exercising *promotive* voice means that employees are speaking up about ways to improve work processes or performance, whereas *prohibitive* voice refers to proposing solutions for decreasing inefficiencies. [36] Both forms of voice serve a purpose, but promotive voice is particularly important for science teams by cultivating a norm of team members building on each other’s ideas with their own knowledge to improve their strategies.

In summary, an awareness of each other’s expertise on a team and effectively sharing information are key for science team effectiveness. Facilitating awareness and exchange can be seen to address teams with diverse backgrounds and skills by making explicit the values, norms, and expectations of all team members. Continued awareness and exchange well-serves science teams that change in composition over time by reducing the misunderstanding of roles and goals. For large teams, and those physically distant and institutionally separate, awareness and exchange can no doubt aid in facilitating coordination and reduction of manifest differences.

### Psychological Safety

A climate of psychological safety is marked by free sharing of ideas, opinions, suggestions, perspectives, and questions in groups without fear of embarrassment, threats to one’s reputation, or retribution of any kind. [37] This is similar to the concept of voice, but differs in that psychological safety is about the team having a shared feeling of freedom in exercising their voice without fear of backlash. Meta-analyses show that teams with high levels of psychological safety are more engaged and committed, as well as have greater team learning, citizenship behaviors, and performance outcomes. [38] Because many science teams are distributed and must collaborate virtually, psychological safety can be difficult to maintain. Researchers have demonstrated the detrimental impact of low psychological safety in their study of virtual teams, finding that teams who perceive a lack of psychological safety were less innovative than other teams. [39] Through examination of the literature, we identify three key factors that can help to support psychological safety: (1) perspective seeking, (2) acknowledging and including others, and (3) addressing issues and resolving conflict.

By seeking the perspectives of others, team members signify an openness to feedback and encourage each other to share their unique viewpoints with the group. One way to seek perspectives of others might be by instituting regular debriefing sessions after performance episodes or at salient points in the project timeline. Debriefs give team members a safe context in which to share ideas and a clearly defined opportunity to seek each other’s perspectives. This goes hand in hand with acknowledging and including those perspectives, which Edmondson [37] names as important for supporting psychological safety. In other words, it is important not only to listen to each other’s ideas, but also to actually work toward implementing them in the project plan. Finally, by addressing issues as they arise rather than waiting for them to fester, teams can limit barriers to psychological safety due to conflict. Resolving conflict, particularly interpersonal conflict, keeps the focus on the objective and performance of the science team.

Psychological safety is beneficial for diverse science teams by ensuring that their interactions and sharing of different perspectives will not be overshadowed by colleagues with more social power. When psychological safety is low, it can be difficult for those with less power or rank to speak up with new ideas, suggestions for improvement, or admissions of errors because they have more to lose when it comes to fear of judgment and threats to reputation. In science teams, junior investigators may yield to more senior members and avoid challenging the status quo, withholding potentially valuable perspectives. Psychological safety is also particularly important in large disparate groups when initial trust can be critical, which is common in multi-institution collaborations. Moreover, a team’s level of psychological safety can be jeopardized by the addition or loss of team members, as levels the team must adapt to changing membership while maintaining a shared feeling that speaking up is accepted and encouraged. By maintaining psychological safety, science teams can ensure that all disciplines, backgrounds, and differing generations can contribute to the scientific enterprise and to the development of the team.

### Adaptation and Self-Correction

Effective science teams can adapt to changing demands and self-correct when necessary. In order to self-correct, teams should be aware of each other’s performance and the progress of the team as a whole toward their goals by monitoring these systems. [40] This might involve practicing a routine of monitoring progress, reflecting on performance, then creating change where it is required. Team debriefing will support these efforts to reflect on and analyze past work and progress toward the team’s goals. The debrief is the most powerful, yet underutilized tool for improving teamwork and team performance. Meta-analyses suggest that teams who debrief outperform other teams by 20%–25%. [41] Debriefing provides a mechanism to head off any confusion or misunderstandings so that team members always have role clarity and maintain aligned efforts, goals, and mental models throughout their collaboration together. They also provide a platform for safely discussing conflicting ideas and creating plans for change and development.

Science teams who effectively adapt and self-correct continually reexamine acquired skills and background of team members, as well as perspectives upon the scientific problem. This is essential for science teams, as its members are consistently evolving with scientific developments in their respective fields. Furthermore, adaptation and correction allow for periodic reexamination of team member goals, thus facilitating coordination and integration across the life cycle of the project. Finally, adaptation and correction allow for the onboarding and socialization for transfer of knowledge over time, as well as maintaining a team development plan. This is critical for addressing the issue of changing membership in science teams, which can occur regularly over the course of a project.

### How TeamMAPPS Addresses Core Team Competencies, Drivers of Team Effectiveness, and Team Science Best Practices

Considering the unique challenges and barriers to science team effectiveness, along with supporting evidence and theory from the general team and science team literature, we identify nine core teamwork competencies that are congruent with contemporary science team requirements and able to be targeted in training and team development interventions. [42] Specifically, we believe these nine competencies impact science team performance by enabling innovation and adaptation through the use of many cognitive, attitudinal, behavioral, and interpersonal skills and abilities in conducting collaborative team research. Given the large number of potential competencies identifiable, [15] no one training program could possibly address all needs. Thus, the question is what might be the most important and amenable to development?

Table 2 showsthe relationship of the nine TeamMAPPS competencies to established general team core competencies, [23] translational team competency domains, [15] identified team effectiveness drivers, [25] and recently developed team science best practices. [43] As illustrated, each of the nine TeamMAPPS competencies can be assessed as high, moderate, or low relatedness, or not applicable. The nine TeamMAPPS competencies fair well across all criteria, particularly in relation to leadership adaptability, cooperation, team communication, creative problem solving, team leadership, coaching, culture of trust, accountability, openness, inclusivity, constant learning, and interdisciplinary conventions. Table 3 details the TeamMAPPS training modules targeting the core competencies alongside action strategies to be learned in training.

**Table 2.** Relationship of TeamMAPPS competencies and general team competencies, effective drivers, and team science best practices

Core Competencies, Drivers of Effectiveness, Best Practices Dimensions	Awareness & Exchange			Psychological Safety			Adaptation & Correction		
	Sharing Unique Info/Promotive Voice (1)	Inquiring/Probing (2)	Reframing & Integrating (3)	Perspective Seeking (4)	Acknowledging & Including (5)	Addressing Issues & Resolving Conflict (6)	Monitoring/Debriefing (7)	Reflecting/Analyzing (8)	Creating Change/Development Plans (9)
Big 5 Core Competencies (Salas, Sims, & Burke, 2005)									
Effective leadership	High	High	High	High	High	High	High	High	High
Mutual performance monitoring	–	–	–	–	–	Moderate	High	High	Low
Backup behavior	–	–	–	–	–	–	High	Low	Low
Adaptability	–	–	High	Low	Low	Moderate	High	Low	High
Team orientation	–	–	Moderate	Moderate	Low	Low	–	Low	High
Transformational Team Competency Domains (Lotrecchiano, DiazGranados, Sprecher, et al., 2021)									
Facilitating team affect	–	–	Low	–	High	–	Low	Low	–
Team communication	High	High	Moderate	High	High	High	Moderate	Low	–
Managing team research	–	–	Low	Low	Moderate	–	Low	–	Moderate
Collaborative problem solving	High	High	Moderate	High	High	Moderate	Moderate	Moderate	Low
Team leadership	High	High	High	High	High	Moderate	Moderate	Moderate	Low
Drivers of Team Effectiveness (Tannenbaum & Salas, 2020)									
Capacity	–	–	–	–	Low	Low	–	Low	Moderate
Cooperation	High	High	Moderate	High	High	High	Low	Moderate	High
Communication	High	High	High	High	High	High	Low	Moderate	Moderate
Cognition	Moderate	Low	Moderate	Low	Low	–	–	Low	–
Coaching	High	High	High	High	High	High	High	High	High
Conditions	Low	Moderate	–	–	High	High	High	–	High
Team Science Best Practices (Rolland, Burnside, Voils, Shah, Brasier, 2020)									
Shared mission, vision, goals	High	High	High	Moderate	–	–	–	Low	High
Culture of trust, accountability, openness, inclusivity, constant learning	High	High	High	High	High	High	Moderate	Moderate	High
Interdisciplinary conversations on approach, methods, results	High	High	Moderate	High	High	Low	Low	Low	Moderate
Information management, coordination, project management, communication system	Moderate	High	Moderate	Moderate	Low	–	High	–	–
Assessing transparent management systems	–	–	–	–	–	–	–	–	–
Strong functional leadership	High	High	High	High	High	High	High	High	High

*Note*: TeamMAPPS = Team Methods to Advance Processes and Performance in Science.

**Table 3.** TeamMAPPS competencies and action strategies

Competencies	Action Strategies
FACILITATING AWARENESS & EXCHANGE • Sharing unique information and promotive voice: sharing information and perspective stemming from expertise, sharing vision of team purpose, building on others’ ideas from our perspective • Inquiring and probing: inquiring to gain information about who knows what on the team, actively probing to create understanding that reflects input of all team members • Reframing and integration: Allowing the input of others to influence personal opinions and ideas, achieving a mutual understanding that reflects the input of all team members	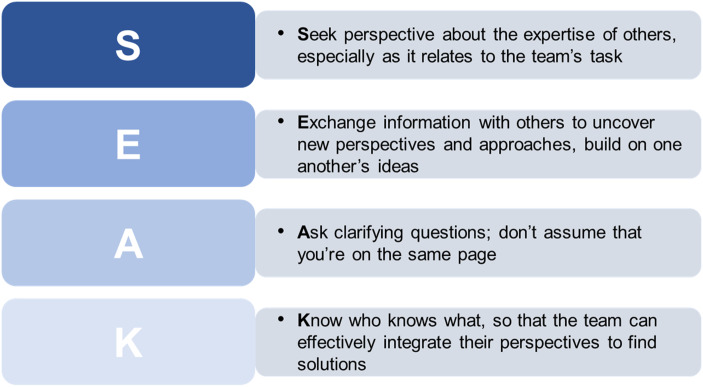
PSYCHOLOGICAL SAFETY • Perspective seeking: asking others about their background, sharing vivid descriptions of experiences, and asking follow-up questions • Acknowledging and including: using closed-loop communication by acknowledging messages and confirming shared understanding, avoiding criticism of new ideas, and matching others’ tasks to their unique skill sets • Addressing issues and resolving conflict: openly discussing mistakes and minimizing interpersonal issues in group interactions	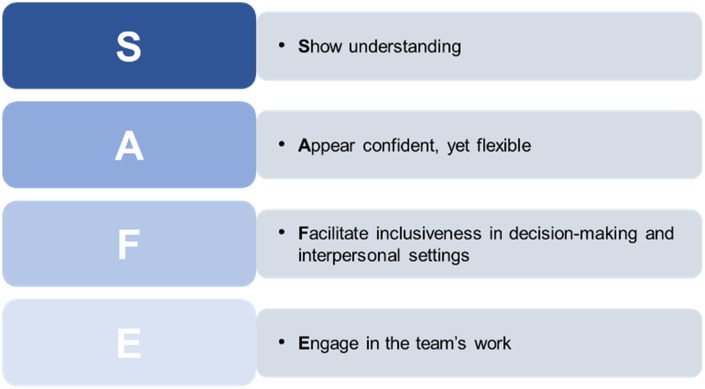
SELF-CORRECTION & ADAPTATION • Monitoring and debriefing: adjusting tasks to accommodate other team members’ needs, discussing performance episodes when completed, documenting incidents • Reflecting and analyzing: reviewing documents and incidents to be discussed, reviewing feedback reports on performance, discussing team strengths and weaknesses among team members • Creating change/development plans: discussing and exploring goals with team members, making needed changes without hesitation, relearning procedures when necessary	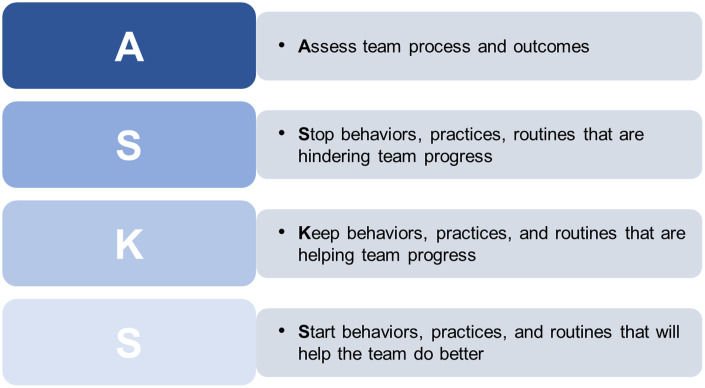

*Note*: TeamMAPPS = Team Methods to Advance Processes and Performance in Science.

### Dissemination, Evaluation, and Development Plans

A distinguishing characteristic of the TeamMAPPS program is its versatility and ability to potentially include assessment, training, and evaluation. Although other valuable development and evaluation systems exist, [44] TeamMAPPS is unique in that it allows users to independently apply some or all components for several purposes such as evaluation, development, and/or assessment for individuals, new teams, or existing teams. To this extent, the different components depicted in Fig. 1 will allow translational team science educators and researchers considerable flexibility in its use. It should be noted, however, that the nine specific competencies for science teams represent only a fraction of the potential competencies to ultimately be identified as predictive of team effectiveness.

The TeamMAPPS program, and its subsequent dissemination, will follow the model of evidence-based interventions, and more specifically one of intervention dissemination. [45] This program is based on research suggesting its components (e.g., psychological safety) have been proven to be effective in team practice. TeamMAPPS will implement a framework that defines variables and the relationships between them. [46] By systematizing the materials and evaluation plan, TeamMAPPS can address numerous problems in implementation such as lack of a common language, inconsistent applications, or lack of embedded evaluation plans. [46] TeamMAPPS will implement specific and behaviorally defined competencies and action plans through systematic content presentation, behavioral models, and exercises. Thus, having reproducible methods [46] can greatly enhance intervention effectiveness. In addition, providing systematic methods and their standardized presentation to trainees will address fidelity concerns, as well as the implementation process gap [47] of quality control across the consortium as a function of variations in organizational capacity.

**Fig. 2.**
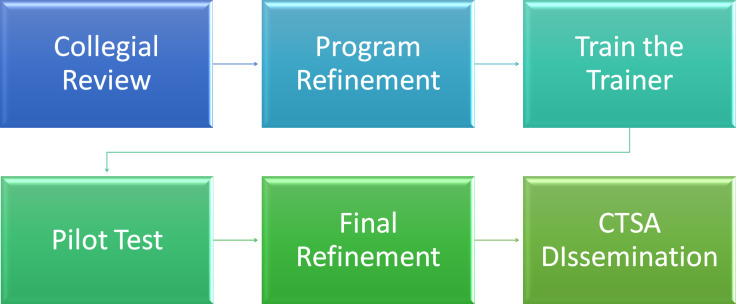
Dissemination plans for TeamMAPPS. *Note*: TeamMAPPS = Team Methods to Advance Processes and Performance in Science.

Leading academic researchers and CTSA thought leaders on team science generated a dissemination strategy after several years of program development involving web-based modules with learning content, self-assessment exercises, behavioral vignettes, application exercises, and application plans (Fig. 2). We will deploy a multiyear, six-stage process to CTSA dissemination. Initially, a cross section of CTS-related team science specialists will conduct a collegial review of each module to ensure fidelity and external validity. After final revisions, representatives from five funded CTSAs will attend a Train-the-Trainer workshop to experience the modules first hand and practice numerous modes of program facilitation. The TeamMAPPS program allows for delivery modalities of (1) completely online asynchronous individual delivery, (2) face-to-face and web-mediated module implementation as part of an existing for-credit class, and (3) implementation of all modules as an intervention for new or existing teams.

Following the Train-the-Trainer event, TeamMAPPS will be piloted (using different delivery modalities) by the five CTSA institutions. Student and facilitator reactions and feedback will be solicited to inform necessary revisions. Areas such as clarity, topic fidelity, application relevance, and navigability will be assessed. In addition, competency-specific knowledge acquisition and self-efficacy will be assessed through pre- and post-training surveys. After final adjustments and formatting, the program will be offered to all CTSA institutions and ultimately to the scientific community, providing each participant with a completion certificate.

#### Evaluation plans

Following the levels of evaluation approach, [48] the TeamMAPPS program will follow a multiyear, multistage plan to ultimately answer the question of whether the training program actually works. Using best practices [49] in the evaluation of team training, the TeamMAPPS evaluation plan will use both experimental and quasi-experimental designs, and multiple levels of assessment. As shown in Table 4, the evaluation plan will assess reactions, learning, and behavior at the individual team level using module-specific pre- and post-training measures.

**Table 4.** Evaluation plan for TeamMAPPS

Level of Evaluation	Method
Reaction	Pre- and post-module self-efficacy items
Learning	Pre- and post-module knowledge comparison quiz
Behavior	Pre- and post-module behavior observation forms
Performance	Comparison of teams on publications, new awards, innovation
Return on investment	Comparison of teams funded, size of subsequent grants, number of new projects initiated by team members

*Note*: TeamMAPPS = Team Methods to Advance Processes and Performance in Science.

Specifically, survey items regarding self-efficacy will assess the degree to which participants believe KSAs have improved and will lead to future success. Knowledge acquisition will be evaluated with items validated to assess mastery of core concepts presented in learning materials. The behavioral markers depicted clearly in Table 1 will be used as criteria for behavioral observational scales and for 360° feedback processes to evaluate performance during training exercises and post-class team meetings. A standardized return-on-investment (ROI) rubric will be developed to evaluate trained and non-trained teams on subsequent funding and new project opportunities, with control variables used to address key factors such as team characteristics and previous funding.

#### Future development

**Fig. 3.**
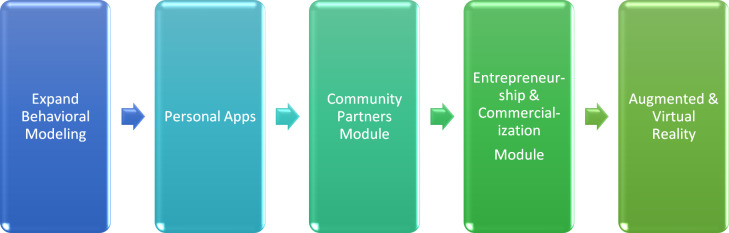
Future development of TeamMAPPS. *Note*: TeamMAPPS = Team Methods to Advance Processes and Performance in Science.

As part of future implementation and dissemination, modifications will be implemented both as a function of evaluative data and of addressing the known needs of team scientists (Fig. 3). We plan to expand the number of behavioral models to maximize transfer of knowledge, [50] provide mobile and web-based applications to personalize the search of specific techniques and action plans needed, provide modules specific to engaging and fully utilizing community members, [4] and entrepreneurial/commercialization experts with scientific teams. Ultimately, we plan to provide augmented and virtual reality exercises to fully contextualize learning opportunities.

## Conclusion

Following a call by the National Research Council [1] to identify and translate KSAs for successful team science, along with a clear need for a team-science-specific competency model and educational curricula, we developed an evidence-based competency model for team scientists – TeamMAPPS. The TeamMAPPS model is valuable for both researchers and practitioners as a framework for designing team training, as well as an intervention diagnostic for assessing teamwork proficiency. Given that there is little evidence or established models of competencies for translational team science, TeamMAPPS is a first stage effort to establish a robust and fully developed taxonomy to use in both professional development and graduate training. Having observable behavior that can be assessed and evaluated provides researchers with a tangible framework to empirically explore and refine. While there exist many potential competencies throughout the team science domain, [15] the three proposed competency sets and their nine attendant specific competencies are useful as much-needed building blocks for the development, validation, and practical application for team science skill development.

## References

[1] National Research Council. Enhancing the Effectiveness of Team Science. Washington, DC: The National Academies Press, 2015. 26247083

[2] Allen NJ, Hecht TD. The ‘romance of teams’: toward an understanding of its psychological underpinnings and implications. Journal of Occupational and Organizational Psychology 2004; 77(4): 439–461.

[3] Collins FS, Wilder EL, Zerhouni E. NIH roadmap/common fund at 10 years. Science 2014; 345(6194): 274–276. 25035478 10.1126/science.1255860 PMC5101933

[4] Tebes JK, Thai ND. Interdisciplinary team science and the public: steps toward a participatory team science. American Psychologist 2018; 73(4): 549–562. 10.1037/amp0000281 PMC5973546 29792467

[5] Jones BF, Wuchty S, Uzzi B. Multi-university research teams: shifting impact, geography, and stratification in science. Science 2008; 322(5905): 1259–1262. 18845711 10.1126/science.1158357

[6] Wuchty S, Jones BF, Uzzi B. The increasing dominance of teams in production of knowledge. Science 2007; 316(5827): 1036–1039. 17431139 10.1126/science.1136099

[7] Begg MD, Crumley G, Fair AM, et al. Approaches to preparing young scholars for careers in interdisciplinary team science. Journal of Investigative Medicine 2014; 62(1): 14–25. 24169319 10.231/JIM.0000000000000021 PMC3970261

[8] Falk-Krzesinski HJ, Börner K, Contractor N, et al. Advancing the science of team science. Clinical and Translation Science Journal 2010; 35(5): 263–266. 10.1111/j.1752-8062.2010.00223.x PMC2965626 20973925

[9] Delise LA, Gorman CA, Brooks AM, Rentsch JR, Steele-Johnson D. The effects of team training on team outcomes: a meta-analysis. Performance Improvement Quarterly 2010; 22(4): 53–80.

[10] Hall KL, Vogel Al, Huang GC, et al. The science of team science: a review of the empirical evidence and research gaps on collaboration in science. American Psychologist 2018; 73(4): 532–548. 10.1037/amp0000319 29792466

[11] Crawford ER, LePine JA. A configural theory of team processes: Accounting for the structure of taskwork and teamwork. Academy of Management Review 2013; 38(1): 32–48.

[12] Disis ML, Slattery JT. 2010. The road we must take: multidisciplinary team science. Science Translational Medicine 2010; 2(22): 22cm9–22cm9. 10.1126/scitranslmed.3000421 20374998

[13] Buljac-Samardzic M, Dekker-van Doorn CM, van Wijngaarden JD, van Wijk KP. Interventions to improve team effectiveness: a systematic review. Health Policy 2010; 94(3): 183–195. 19857910 10.1016/j.healthpol.2009.09.015

[14] Börner K, Contractor N, Falk-Krzesinski HJ, et al. A multi-level systems perspective for the science of team science. Science Translational Medicine 2010; 2(49): 49cm24. 10.1126/scitranslmed.3001399 PMC3527819 20844283

[15] Lotrecchiano GR, DiazGranados D, Sprecher J, et al. Individual and team competencies in translational teams. Journal of Clinical and Translational Science 2020; 1–20. doi: 10.1017/cts.2020.551. PMC8057415 33948290

[16] Salas E, DiazGranados D, Klein C, et al. Does team training improve team performance? A meta-analysis. Human Factors 2008; 50(6): 903–933. 19292013 10.1518/001872008X375009

[17] Xia L, Ya S. Study on knowledge sharing behavior engineering. Systems Engineering Procedia 2012, 4: 468–476.

[18] Eisenbeiss SA, Van Knippenberg D, Boerner S. Transformational leadership and team innovation: integrating team climate principles. Journal of Applied Psychology 2008; 93(6): 1438. 10.1037/a0012716 19025260

[19] Wooten KS, Calhoun WJ, Bhavnani S, Rose RM, Ameredes B, Brasier AR. Evolution of multidisciplinary translational teams (MTTs): insights for accelerating translational innovations. Clinical and Translational Science 2015; 8(5): 542–552. 25801998 10.1111/cts.12266 PMC4575623

[20] Chao C, Wooten K, Spratt H, et al. Integration of leadership training for graduate and medical students engaged in translational biomedical research: Examining self-efficacy and self-insight. Journal of Clinical and Translational Science 2018; 2(1): 48–52. 31660217 10.1017/cts.2018.9 PMC6799644

[21] Bisbey TM, Reyes DL, Traylor AM, Salas E. Teams of psychologists helping teams: the evolution of the science of team training. American Psychologist 2019; 74(3): 278–289. 10.1037/amp0000419 30945891

[22] Stevens MJ, Campion MA. The knowledge, skill, and ability requirements for teamwork: implications for human resource management. Journal of Management 1994; 20(2): 503–530.

[23] Salas E, Sims De, Burke CS. Is there a “Big Five” in Teamwork? Small Group Research 2005; 36(5): 555–599.

[24] Salas E, Rosen MA, Burke CS, Goodwin GF. The wisdom of collectives in organizations: an update of the teamwork competencies. In: Salas E, Goodwin GF, Burke CS, eds. The Organizational Frontiers Series. Team Effectiveness in Complex Organizations: Cross-disciplinary Perspectives and Approaches. New York, NY: Routledge, 2009, pp. 37–79.

[25] Tannenbaum S, Salas E. Teams That Work: The Seven Drivers of Team Effectiveness. New York, NY: Oxford University Press, 2020.

[26] Clancy CM, Tornberg DN. TeamSTEPPS: assuring optimal teamwork in clinical settings. American Journal of Medical Quality 2007; 22(3): 214–217. 17485563 10.1177/1062860607300616

[27] King HB, Battles J, Baker DP, et al. TeamSTEPPS™: team strategies and tools to enhance performance and patient safety. In: Henriksen K, Battles JB, Keyes MA, Grady ML, eds, Advances in Patient Safety: New Directions and Alternative Approaches (Vol. 3: Performance and Tools). Rockville, MD, 2008.

[28] Agency for Healthcare Research and Technology (AHRQ). (2015, July). *TeamSTEPPS®: Research/Evidence Base. Agency for Healthcare Research and Quality* [Internet], July 2015 [cited Feb 8, 2021]. (https://www.ahrq.gov/teamstepps/evidence-base/index.html)

[29] Gebbie KM, Mason Meier B, Bakken S, et al. Training for interdisciplinary health research defining the required competencies. Journal of Allied Health 2008; 37(2): 65–70. 18630780

[30] Hall KL, Vogel AL, Stipelman BA, Stokols D, Morgan G, Gehlert S. A four-phase model of transdisciplinary team-based research: goals, team processes, and strategies. Translational Behavioral Medicine 2012; 2(4): 415–430. 23483588 10.1007/s13142-012-0167-y PMC3589144

[31] Shuffler ML, Jimenez-Rodriguez M, Kramer WS. The science of multiteam systems: a review and future research agenda. Small Group Research 2015; 46: 659–699.

[32] Vogel AL, Hall Kl, Fiore SM , et al. The team science toolkit: enhancing research collaboration through online knowledge sharing. American Journal of Preventative Medicine 2013; 46(6): 787–789. 10.1016/j.amepre.2013.09.001 24237924

[33] Austin JR. Transactive memory in organizational groups: The effects of content, consensus, specialization, and accuracy on group performance. Journal of Applied Psychology 2003; 88: 866–878. doi: 10.1037/0021-9010.88.5.866. 14516250

[34] Salazar M, Lant TK, Fiore SM, Salas E. Facilitating innovation in diverse science teams through integrative capacity. Small Group Research 2012; 43(5): 527–558.

[35] Van Der Vegt GS, Bunderson JS. Learning and performance in multidisciplinary teams: the importance of collective team identification. Academy of Management Journal 2005; 48(3): 532–547.

[36] Liang J, Farh CI, Farh JL. Psychological antecedents of promotive and prohibitive voice: a two-wave examination. Academy of Management Journal 2012; 55(1): 71–92.

[37] Edmondson A. Psychological safety and learning behavior in work teams. Administrative Science Quarterly 1999; 44(2): 350–383.

[38] Frazier ML, Fainschmidt S, Klinger RL, Pezeshkan A, Vracheva V. Psychological safety: A meta-analytic review and extension. Personnel Psychology 2017; 70: 113–165. doi: 10.1111/peps.12183.

[39] Gibson CB, Gibbs JL. Unpacking the concept of virtuality: The effects of geographic dispersion, electronic dependence, dynamic structure, and national diversity on team innovation. Administrative Science Quarterly 2006; 51: 451–495. doi: 10.2189/asqu.51.3.451.

[40] LePine JA. Team adaptation and post change performance: effects of team composition in terms of members’ cognitive ability and personality. Journal of Applied Psychology 2003; 88(1): 27. 10.1037/0021-9010.88.1.27 12675392

[41] Tannenbaum SI, Cerasoli CP. Do team and individual debriefs enhance performance? A meta-analysis. Human Factors 2013; 55(1): 231–245. 23516804 10.1177/0018720812448394

[42] Wooten K, Salas E, Lant T, et al. An evidence-based competency model for team science training. In: *Poster presented at the 8th Annual International Science of Team Science Conference*. Ft. Lauderdale, FL: May 2017.

[43] Rolland B, Burnside ES, Voils CI, Shah MN, Brasier AR. Enhancing reproducibility using interprofessional team best practices. Journal of Clinical and Translational Science 2020; 1–8. doi: 10.1017/cts.2020.512. PMC8057443 33948243

[44] Colorado State University. *Team Development and Evaluation: Developmental Evaluation* [Internet], 2021 [cited Apr 21, 2021]. (https://www.research.colostate.edu/cctsi/programs-services/team-science/)

[45] Rabin BA, Brownson RC, Haire-Joshu D, Kreuter MW, Weaver NL. A glossary for dissemination and implementation research in health. Journal of Public Health Management and Practice 2008; 14(2): 117–123. 18287916 10.1097/01.PHH.0000311888.06252.bb

[46] Rapport F, Clay-Williams R, Churruca K, Shih P, Hogden A, Braithwaite J. The struggle of translating science into action: foundational concepts of implementation science. Journal of Evaluation in Clinical Practice 2018; 24(1): 117–126. 28371050 10.1111/jep.12741 PMC5901403

[47] Bauer MS, Damschroder L, Hagedorn H, Smith J, Kilbourne AM. An introduction to implementation science for the non-specialist. BMC Psychology 2015; 3(1): 1–12. 26376626 10.1186/s40359-015-0089-9 PMC4573926

[48] Kirkpatrick D, Kirkpatrick J. Evaluating Training Programs: The Four Levels. Oakland, CA: Berrett-Koehler Publishers, 2006.

[49] Weaver SJ, Salas E, King HB. Twelve best practices for team training evaluation in health care. The Joint Commission Journal on Quality and Patient Safety 2011; 37(8): 341–349. 21874969 10.1016/s1553-7250(11)37044-4

[50] Taylor PJ, Russ-Eft DF, Chan DW. A meta-analytic review of behavior modeling training. Journal of Applied Psychology 2005; 90(4): 692. 10.1037/0021-9010.90.4.692 16060787

